# Thrombin Maybe Plays an Important Role in MK Differentiation into Platelets

**DOI:** 10.1155/2016/9313269

**Published:** 2016-03-15

**Authors:** Xiao-Lei Yang, Meng-Kai Ge, De-Kui Mao, Ying-Tao Lv, Shu-Yan Sun, Ai-Ping Yu

**Affiliations:** ^1^Department of Experimental Hematology, Beijing Institute of Radiation Medicine, Beijing 100850, China; ^2^College of Chemical Engineering, Qingdao University of Science and Technology, Qingdao 266042, China; ^3^The 107th Hospital of PLA and The Affiliated Hospital of Bin Zhou Medical University, Yantai 264000, China

## Abstract

*Objectives*. After development and differentiation, megakaryocytes (MKs) can produce platelets. As is well known, thrombopoietin (TPO) can induce MKs to differentiate. The effect of thrombin on MKs differentiation is not clear. In this study, we used a human megakaryoblastic leukemia cell line (Meg-01) to assess the effect of thrombin on MKs differentiation.* Methods*. In order to interrogate the role of thrombin in Meg-01 cells differentiation, the changes of morphology, cellular function, and expression of diverse factors were analyzed.* Results*. The results show that thrombin suppresses Meg-01 cells proliferation and induces apoptosis and cell cycle arrest. Thrombin upregulates the expression of CD41b, which is one of the most important MK markers. Globin transcription factor 1 (GATA-1), an important transcriptional regulator, controls MK development and maturation. The expression of GATA-1 is also upregulated by thrombin in Meg-01 cells. The expression of B-cell lymphoma 2 (Bcl-2), an apoptosis-inhibitory protein, is downregulated by thrombin. Phosphorylated protein kinase B (p-AKT) and phosphorylated extracellular signal-regulated kinase (p-ERK) were upregulated by thrombin in Meg-01 cells. All the results are consistent with Meg-01 cells treated with TPO.* Discussion and Conclusion*. In conclusion, all these data indicate that thrombin maybe plays an important role in MK differentiation into platelets. However, whether the platelet-like particles are certainly platelets remains unknown.

## 1. Introduction

Thrombin is one of the most important blood coagulation factors. As an important mitogen, thrombin induces many cellular functions. Protease-activated receptors (PARs) are the main receptors of thrombin, and PAR1 and PAR4 are distributed on the membrane of platelets and megakaryocytes (MKs). Thrombin activates platelets mainly through PAR1 and PAR4, and after treatment with thrombin, platelets undergo the processes of shape change, adhesion, aggregation, and secretion [1, 2]. Considering that platelets are produced from MKs and PARs are expressed in MKs, we presumed that thrombin/PARs may exert some role in MK differentiation into platelets.

At present, there are two main theories about platelet production from MKs: the “proplatelet theory” [3] and the “explosive-fragmentation theory” [4]. The “proplatelet theory” was proposed in recent years. Proplatelets are made by mature MKs and develop into platelets through extension, amplification, and moving near to the bone marrow venous sinusoids [5].

The megakaryoblastic leukemia cell line (Meg-01) was established by Ogura et al. in 1985 [6]. It displays phenotypic properties that closely resemble those of MKs. Some research has shown that the Meg-01 cell line produces functional platelets under normal culture conditions after exposure to aphidicolin [1]. In this study, we used Meg-01 as model cells to elucidate the effects of thrombin on cell differentiation into platelets and the corresponding mechanism. As thrombopoietin (TPO) is a crucial growth regulator in the differentiation of MKs and platelet production, we chose TPO as a positive control in this study.

## 2. Materials and Methods

### 2.1. Reagents

Bovine thrombin and bovine serum albumin (BSA) were purchased from Sigma (St. Louis, MO, USA). Mouse anti-p-ERK, rabbit anti-ERK, goat anti-AKT1/2, and rabbit anti-p-AKT1 antibodies were purchased from Santa Cruz Biotechnology (Santa Cruz, CA, USA). Rabbit anti-CD41 antibody was obtained from Abcam (Cambs., UK). Horseradish peroxidase- (HRP-) labeled goat anti-mouse immunoglobulin G (IgG), goat anti-rabbit IgG, and rabbit anti-goat IgG antibodies were obtained from Jackson Immuno Research (West Grove, PA, USA). R-phycoerythrin- (R-PE-) conjugated goat anti-rabbit IgG (H + L) was acquired from Proteintech (Chicago, USA). Fluorescein isothiocyanate- (FITC-) conjugated anti-CD61 antibody was purchased from eBioscience (CA, USA).

### 2.2. Cell Culture

The Meg-01 cell line was obtained from the Kunming Cell Bank of the Chinese Academy of Sciences (Kunming, China). Roswell Park Memorial Institute 1640 (RPMI-1640) medium was acquired from Invitrogen (Gibco, CA, USA). The cells were cultured in RPMI-1640 medium containing 10% FBS and cells were incubated in saturated humidity at 37°C with 5% CO_2_.

### 2.3. Microscopy and Analysis

To evaluate the effect of thrombin on Meg-01 cell morphology, live cells were observed by light microscopy (OLYMPUS, Japan). The old media were removed and the cells were washed with PBS prior to imaging. Pictures were captured with a camera system.

### 2.4. Viability Assay

Cell viability was assessed by a cell counting kit-8 (CCK-8) assay (Dojindo, Kumamoto, Japan). Meg-01 cells (1 × 10^5^ cells/well) were plated into 96-well plates. After 8 h, the cells were treated with thrombin (final concentration = 2 U/mL) and incubated for 24 h. After this time, the CCK-8 reagent was added to each well and mixed well, and then cells were incubated for 2 h at 37°C. The absorbance at 450 nm was measured. Viable particles were measured by an AlamarBlue cell viability assay kit (KeyGEN BioTECH, Nanjing, China). TPO (80 ng/mL) was used as a positive control.

### 2.5. Quantitative Real-Time PCR Assay

After the cells were treated with thrombin (final concentration = 2 U/mL) for 24 h, total RNA was extracted from Meg-01 cells withanEasy Pure*™* RNA kit (TransGen Biotech, Beijing, China). The reverse transcription reaction was executed using 1 *µ*g of total RNA, which was reverse-transcribed into cDNA with TransScript First-Strand cDNA Synthesis SuperMix (TransGen Biotech, Beijing, China). Quantitative real-time PCR (qPCR) analysis was carried out with TransStart Top Green qPCR SuperMix (TransGen Biotech, Beijing, China). The mRNA expression was analyzed by an ABI 7500 real-time PCR system (Applied Biosystems, CA, USA). The primer sequences used were as follows: GATA-1, forward 5′-CAGTAAACGAGCAGGTACTC-3′ and reverse 5′-CATAAAGCCACCAGCTGGTC-3′; Bcl-2, forward 5′-GTGGAGGAGCTCTTCAGGGA-3′ and reverse 5′-AGGCACCCAGGGTGATGCAA-3′; GAPDH, forward 5′-GGATTTGGTCGTATTGGG-3′ and reverse 5′-TCGCTCCTGGAAGATGG-3′. qPCR assays were carried out in triplicate. Relative gene expression was obtained after normalization with GAPDH and followed by comparison to the control.

### 2.6. Western Blot Analysis

Cellular lysates were prepared. Target proteins were resolved using sodium dodecyl sulfate polyacrylamide gel electrophoresis (SDS-PAGE) and transferred to Immobilon PVDF membranes. The membranes were blocked with 3% BSA for 2 h at room temperature, probed with antibodies (1 : 1000) overnight at 4°C, and washed with tris-buffered saline with Tween 20 (TBST) prior to incubation with secondary antibodies (1 : 5000). Specific antibody binding was detected by chemiluminescence.

### 2.7. Apoptosis Analysis by Flow Cytometry

The percentage of apoptotic cells was detected using an Annexin V FITC apoptosis detection kit (KeyGEN BioTECH, Nanjing, China), according to the manufacturer's protocol. After thrombin (2 U/mL) or TPO (80 ng/mL) treatment, cells were collected and washed with phosphate-buffered saline (PBS). Cells were stained in the dark for 15 min at room temperature and analyzed by flow cytometry within 1 h of finishing the staining step. Data were analyzed by Analysis Software FlowJo 7.6.1.

### 2.8. CD41 Analysis by Flow Cytometry

After treatment with thrombin (2 U/mL) or TPO (80 ng/mL) for 24 h, cells were harvested in centrifuge tubes and washed with PBS. Cells were incubated with anti-CD41b antibody (1 : 100) in a final volume of 100 *µ*L for 30 min at room temperature. After washing, phycoerythrin- (PE-) conjugated goat anti-rabbit IgG secondary antibody (1 : 50) was added. Then, the centrifuge tubes were placed in the dark for 30 min at room temperature. Cells were analyzed by flow cytometry. Data were analyzed by Analysis Software FlowJo 7.6.1.

### 2.9. Particle Function Assay

Meg-01 cells were incubated with thrombin (final concentration = 2 U/mL) and TPO (final concentration = 80 ng/mL) at 37°C for 24 h. The cell suspension was centrifuged at 1500 rpm for 15 min and the supernatant was harvested and washed with PBS twice. To analyze whether the particles (0.3 × 10^6^ to 1.8 × 10^6^) produced by MKs after thrombin or TPO treatment were active, AlamarBlue was added to the supernatant; then the mixture was incubated for 24 h at 37°C. The absorbance at 570 nm and 600 nm was measured. Different optical density (OD) values were recorded.

To further test whether the particles were functional, the expression of CD61 in the particles was assayed. After treatment with thrombin or TPO for 24 h, particles were harvested in centrifuge tubes and washed with PBS. Particles were incubated with anti-CD61 antibody (1 : 100) to a final volume of 100 *μ*L for 30 min at room temperature in the dark. After washing, particles were analyzed by flow cytometry. Data were analyzed by Analysis Software FlowJo 7.6.1.

### 2.10. Statistical Analysis

All data were described as mean ± standard deviation (SD) and analyzed by Student's *t*-test and one-way analysis of variance. A *P* value of less than 0.05 or 0.01 was considered to be statistically significant.

## 3. Results

### 3.1. Meg-01 Is an MK Cell Line and CD61 Is a Special Marker of MKs

**Figure 1:**
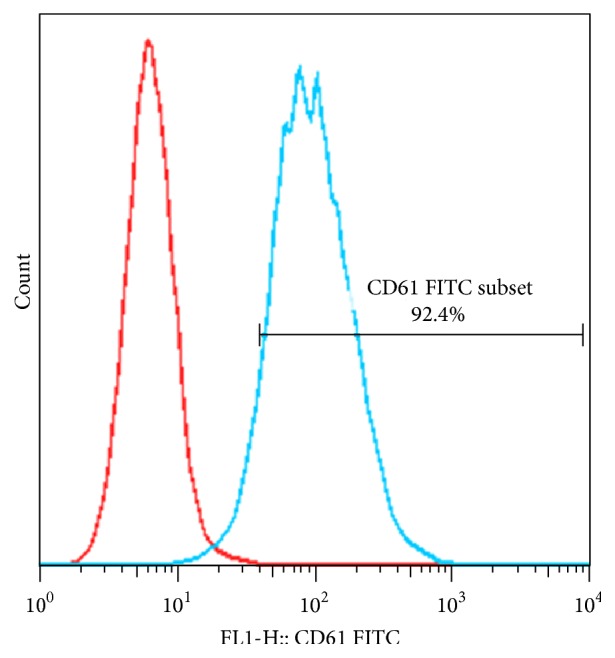
Meg-01 is a megakaryocytic cell line. The expression of CD61 was assayed by flow cytometry (left curves: isotype controls; right curves: antibodies).

To identify the Meg-01 cells used in this study, the expression of CD61 was assayed by flow cytometry. As is shown in Figure 1, the expression of CD61 was up to 92.4% in Meg-01 cells.

### 3.2. Thrombin Changes the Morphology of Meg-01 Cells

**Figure 2:**
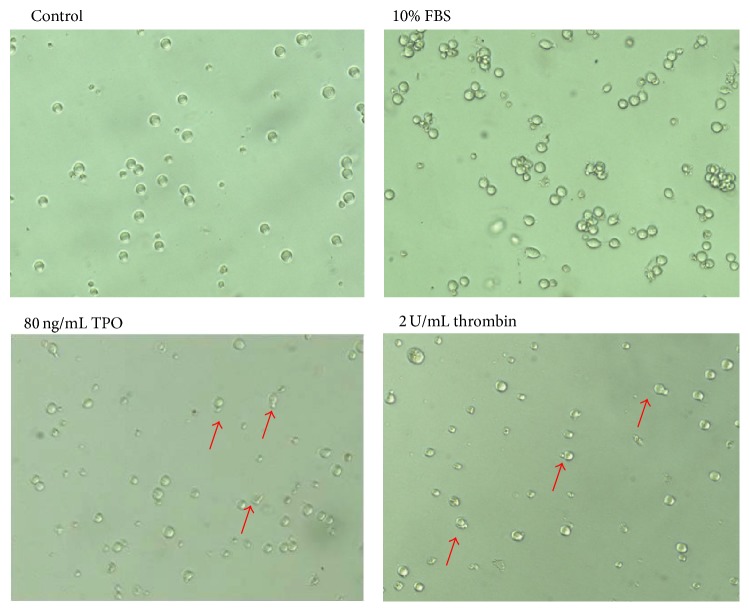
Thrombin promotes the appearance of pseudopodia in Meg-01 cells. After treatment with TPO (80 ng/mL) or thrombin (2 U/mL) for 24 h, cells developed pseudopodia (indicated by arrows). Meanwhile, cells cultured in medium with no treatment (negative control) did not show this phenomenon. Cells cultured in medium containing 10% FBS stuck together. Similar results were observed in two additional experiments.

To analyze the effect of thrombin on cell morphology, Meg-01 cells were treated with thrombin (2 U/mL) for 24 h and the results were compared with cells treated with TPO (80 ng/mL). As is shown in Figure 2, thrombin induced the appearance of pseudopodia in a similar manner to those cells treated with TPO. However, the cells stuck together after treatment with 10% fetal bovine serum (FBS) and the shape of the cells in the control group was regular. After treatment with 10% FBS, the percentage of cells developing pseudopodia is 6.6%. However, in those cells treated with thrombin and TPO, the percentages are 30.4% and 29.6%, respectively.

### 3.3. Thrombin Inhibits Cell Viability and Induces G0/G1 Arrest and Apoptosis in Meg-01 Cells

**Figure 3:**
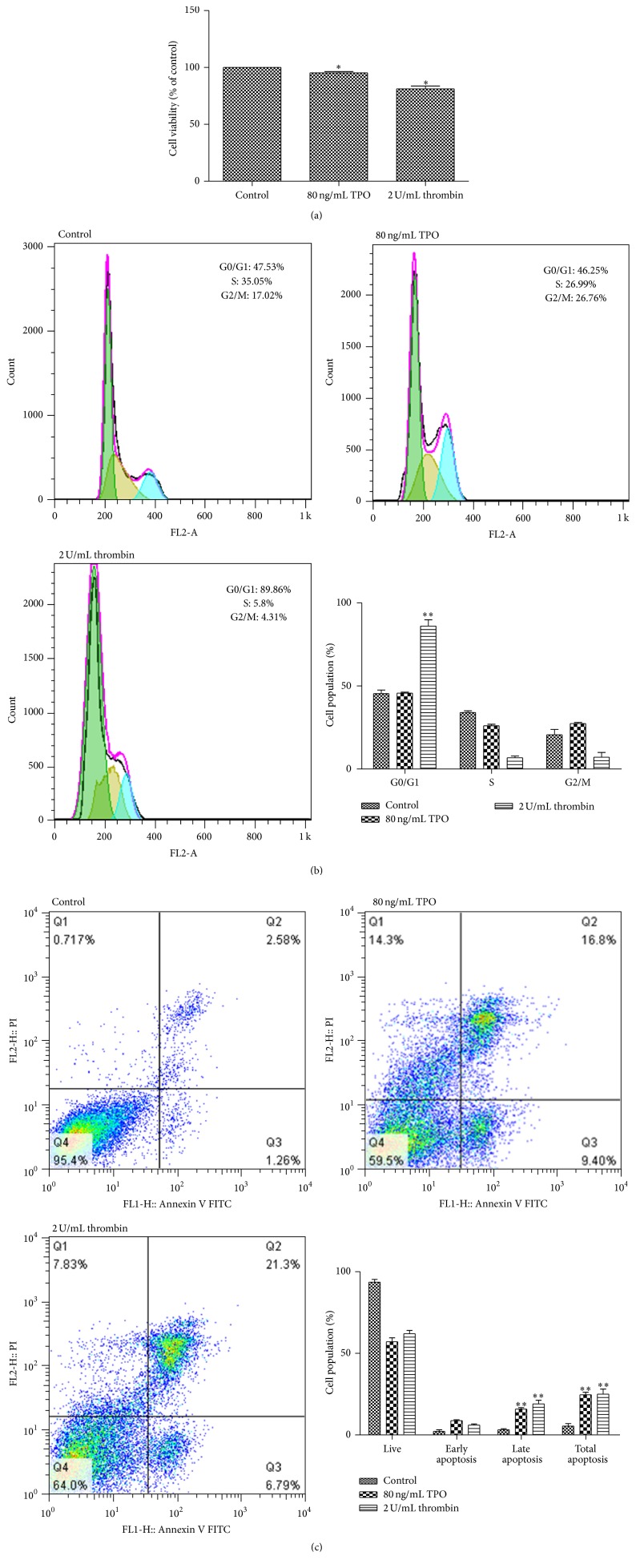
Thrombin inhibits cell proliferation and induces G0/G1 arrest and apoptosis in Meg-01 cells. (a) Meg-01 cell proliferation was assayed by CCK-8. Meg-01 cells were treated with 80 ng/mL TPO or 2 U/mL thrombin for 24 h. (b) After treatment with 80 ng/mL TPO or 2 U/mL thrombin for 24 h, the cell cycle distribution of Meg-01 cells was determined by flow cytometry. (c) The percentage of apoptotic cells was detected via Annexin V/PI staining after cells were treated with thrombin or TPO for 24 h. The data show mean ± SD from three independent experiments. ^*∗*^ *P* < 0.05 and ^*∗∗*^ *P* < 0.01, compared with the control.

To evaluate the effect of thrombin on cell proliferation, Meg-01 cells were treated with thrombin (2 U/mL) for 24 h and the results show that thrombin (2 U/mL) and TPO (80 ng/mL) suppressed cell viability (Figure 3(a)). To further examine the mechanism of thrombin with regard to the inhibition of cell viability, the effects of thrombin on the cell cycle were assessed (Figure 3(b)). The results showed that thrombin (2 U/mL) treatment for 24 h led to G0/G1 phase arrest. To further determine if thrombin had an effect on the apoptosis of Meg-01 cells, flow cytometry analysis was performed. The results showed that cells treated with thrombin (2 U/mL) underwent apoptosis at notably higher rates than the control. TPO (80 ng/mL) also significantly induced apoptosis of Meg-01 cells (Figure 3(c)).

### 3.4. Thrombin Upregulates the Expression of Globin Transcription Factor 1 and Downregulates the Expression of B-Cell Lymphoma 2

**Figure 4:**
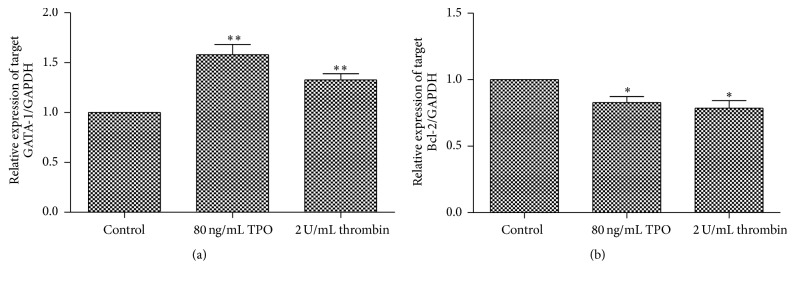
Thrombin influences the expression of GATA-1 and Bcl-2 in Meg-01 cells. GATA-1 (a) and Bcl-2 (b) mRNA levels were quantified by real-time PCR. The data are shown as mean ± SD from three independent experiments. ^*∗*^ *P* < 0.05 and ^*∗∗*^ *P* < 0.01, compared with the control.

To examine the expression of globin transcription factor 1 (GATA-1) and B-cell lymphoma 2 (Bcl-2), quantitative real-time polymerase chain reaction (PCR) was used. The results showed that thrombin (2 U/mL) and TPO (80 ng/mL) upregulated the expression of GATA-1 and downregulated the expression of Bcl-2 (Figure 4).

### 3.5. Thrombin Upregulates the Expression of CD41b

**Figure 5:**
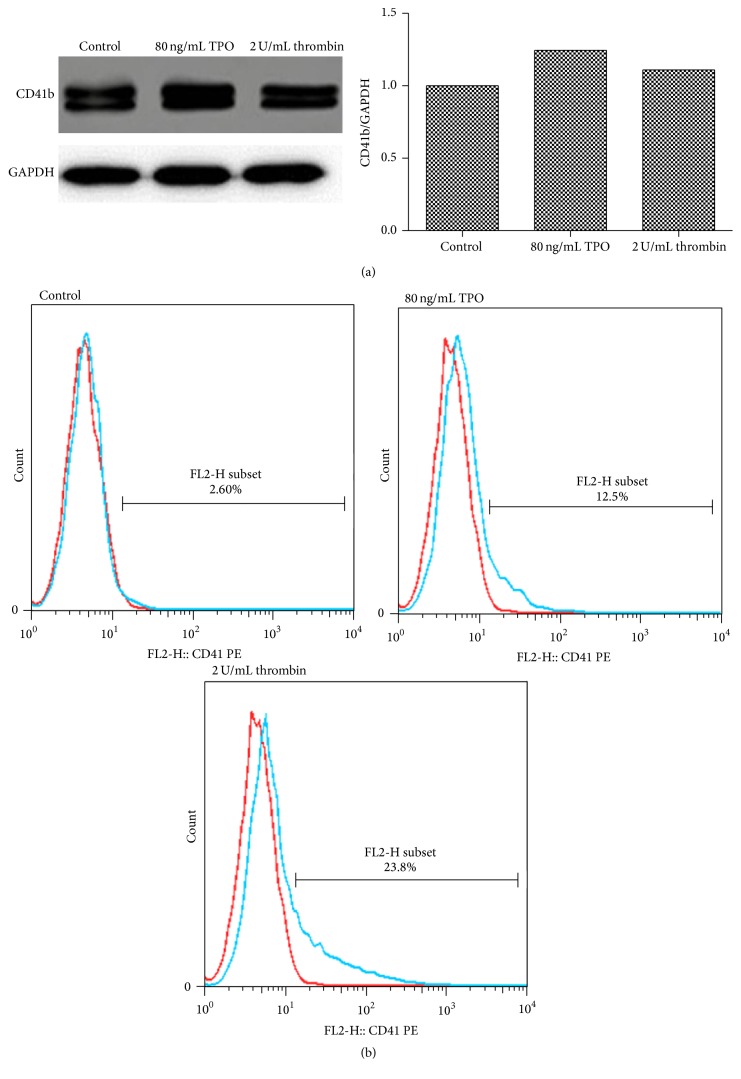
Thrombin upregulates the expression of CD41b in Meg-01 cells. After 8 h incubation, Meg-01 cells were treated with TPO (80 ng/mL) or thrombin (2 U/mL) for 24 h. (a) The expression of CD41b was assayed by western blot. (b) The expression of CD41b was assayed by flow cytometry (left curves: isotype controls; right curves: antibodies).

To estimate the effect of thrombin on Meg-01 cell differentiation, the expression of CD41b was assayed by western blot and flow cytometry. As is shown in Figure 5, both thrombin (2 U/mL) and TPO (80 ng/mL) upregulated the expression of CD41b.

### 3.6. Thrombin Activates the Mitogen-Activated Protein Kinase/Extracellular Signal-Regulated Kinase and the Phosphoinositide 3-Kinase/Protein Kinase B Pathways in Meg-01 Cells

**Figure 6:**
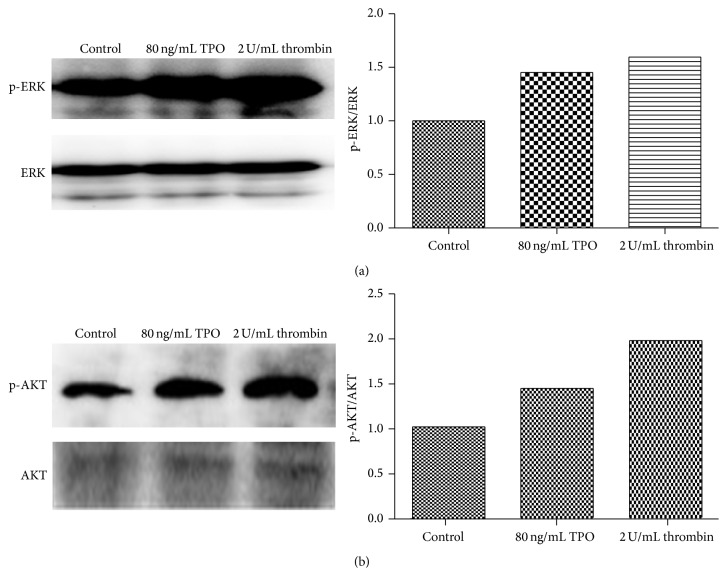
Thrombin activates the MAPK/ERK and PI3K/AKT pathways. After 8 h incubation, Meg-01 cells were treated with TPO (80 ng/mL) or thrombin (2 U/mL) for 24 h. (a) The expression of p-ERK was assayed by western blot. (b) The expression of p-AKT was assayed by western blot.

Mitogen-activated protein kinase/extracellular signal-regulated kinase (MAPK/ERK) and phosphoinositide 3-kinase/protein kinase B (PI3K/AKT) signaling pathways regulate cell differentiation and growth. To investigate the molecular mechanism by which thrombin affects Meg-01 cells, phosphorylation of ERK (p-ERK) and phosphorylation of AKT (p-AKT) were assayed by western blot. The results (Figure 6) showed a significant increase in p-ERK and p-AKT in the thrombin and TPO groups.

### 3.7. Particles Released from Meg-01 Cells after Thrombin Treatment Are Functional

**Figure 7:**
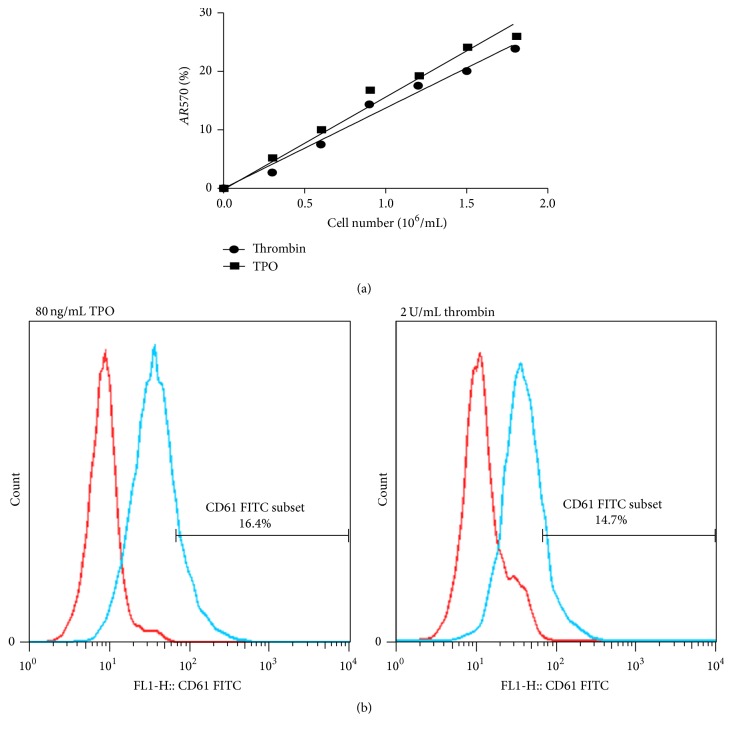
Particles released from Meg-01 cells after thrombin treatment are functional. Meg-01 cells were treated with thrombin (2 U/mL) or TPO (80 ng/mL) for 24 h; then the particles were concentrated by centrifugation. (a) Particles were incubated with AlamarBlue for 24 h at 37°C. The absorbance at 570 nm and 600 nm was measured. Each point represents mean ± SD of three replications. *AR*570 (%) = [*A*570 − (*A*600 × *R*0)] × 100, where *R*0 = *A*570/*A*600. (b) The expression of CD61 in platelet-like particles was assayed by flow cytometry (left curves: isotype controls; right curves: antibodies).

To investigate whether the particles released from Meg-01 cells by thrombin treatment were functional, the particles were collected by centrifugation. The AlamarBlue method was used to detect the viability of the particles [1]. As is shown in Figure 7(a), the absorbance was proportional to the number of particles. This result demonstrated that the particles were viable. To further analyze whether the particles have the functions of platelets, the expression of CD61 in the platelet-like particles was assayed. As is shown in Figure 7(b), the expression of CD61 in the particles released from Meg-01 cells following thrombin or TPO treatment was 14.7% and 16.4%, respectively.

## 4. Discussion

### 4.1. Matured Polyploid MKs Are the Mother Cells of Platelets

During the process of the production of platelets, MKs undergo a morphological change. Some studies have found that platelets are released before the extension of proplatelet-like expansions from the cytoplasm. During differentiation, MKs extend long filamentous projections [7, 8]. In this study, Meg-01 cell produced pseudopodia after exposure to thrombin and TPO (Figure 2) in a process that was similar to the production of platelets from MKs. We consequently presumed that the pseudopodia induced by thrombin stimulation were associated with Meg-01 cell differentiation into platelets.

### 4.2. The Production of Platelets Is Linked to MK Apoptosis

Apoptosis is critical to both the formation of proplatelets and the production of functional platelets [9]. Stimulated by thrombin, Meg-01 cells appear to have apoptosis (Figure 3(c)). In addition, the* Bcl-2*/*Bcl-2-associated X protein* (*BAX*) gene family regulates the apoptotic death signals. In particular, Bcl-2 and Bcl-X_L_ are apoptosis-inhibitory proteins [10–12]. It has been reported that the overexpression of Bcl-2 in CD34 progenitor cells cultured* in vitro* inhibits proplatelet extensions [13] and Bcl-2 is absent from mature blood platelets [14]. In our work, the expression of Bcl-2 was significantly reduced when Meg-01 cells were treated with thrombin (Figure 4). Thrombin exerts similar effects on Meg-01 cell apoptosis and Bcl-2 expression as TPO. We presumed that the production of pseudopodia was linked with the apoptosis of Meg-01 cells and that the reduction of Bcl-2 expression may lead to this apoptosis.

During MK differentiation, GATA-1 is an important transcriptional regulator that controls MK cytoplasmic maturation and development of platelet organelles. Furthermore, GATA-1 may be associated with proplatelet formation and regulation of platelet size and numbers. The expression of GATA-1 is high in erythrocytes, MKs, and rhabdocytes [15–18]. In this study, both thrombin and TPO upregulated the expression of GATA-1 (Figure 4), indicating that they maybe play a role in the maturation and differentiation of Meg-01 cells. CD41 is one of the most important MK markers, which regulates cell adhesion and platelet aggregation by binding fibrinogen and von Willebrand factor [19]. As the degree of MK differentiation rises, CD41 expression increases. Our results show that thrombin and TPO apparently upregulate the expression of CD41b (Figure 5), indicating that thrombin might participate in Meg-01 cell maturation and differentiation.

All the above results predict that thrombin maybe plays an important role in Meg-01 cell maturation and differentiation. However, whether the molecular mechanism is consistent with the mechanism of Meg-01 cell maturation and differentiation must be considered.

### 4.3. The MAPK/ERK Pathway Promotes Differentiation in MKs

ERK-dependent MK differentiation is responsive to TPO [20, 21]. ERK1 and ERK2 are activated after MKs are exposed to TPO and ERK1 and ERK2 have been found to support endomitosis in MKs [22]. ERK regulates the CD41 promoter by combinatorial interactions of nuclear factors that synergize in CD41 promoter regulation [23]. TPO can also activate the PI3K/AKT pathway in primary MKs [24]. Many studies have suggested that several other hematopoietic cytokines, including interleukin 3 (IL-3; [25, 26]), erythropoietin (EPO; [27, 28]), and IL-6 [29], can activate PI3K and AKT. We presumed that the activation of the ERK and PI3K/AKT pathways may relate to the differentiation of MKs. In this study, we found that thrombin can upregulate the expression of p-ERK and p-AKT (Figure 6). This indicated that the two signaling pathways may participate in the process of thrombin induced MK differentiation.

Furthermore, the particles produced by Meg-01 cells after thrombin treatment expressed CD61, which is expressed in platelets, and the particles were viable (Figure 7). This indicated that thrombin induced Meg-01 cells to produce functional platelet-like particles.

All these results indicate that thrombin may play an important role in MK differentiation into platelets. However, whether the platelet-like particles are certainly platelets remains unknown. Comparing the platelet-like particles with normal platelets is currently ongoing in our laboratory.

## Additional Points

In this study, we investigated the effect of thrombin on the Meg-01 cell line. We found that thrombin maybe plays an important role in Meg-01 cell differentiation into platelets.
